# Aneurysmal bone cyst of the pelvis and sacrum: a single-center study of 17 cases

**DOI:** 10.1186/s12891-022-05362-1

**Published:** 2022-04-30

**Authors:** Niklas Deventer, Tymoteusz Budny, Georg Gosheger, Marieke de Vaal, Jana Burkhardt, Nils Deventer

**Affiliations:** 1 Department of Orthopedics and Tumororthopedics, University Hospital Muenster, Albert-Schweitzer-Campus 1, 48149 Muenster, Germany; 2 Department of General Paediatrics, University Children’s Hospital Muenster, Albert-Schweitzer-Campus 1, 48149 Muenster, Germany

**Keywords:** Aneurysmal bone cyst, Polidocanol injection, Polidocanol instillation

## Abstract

**Background:**

The aneurysmal bone cyst (ABC) is a benign, but locally aggressive cystic lesion of the bone. It usually occurs in the metaphysis of long bones of adolescents and young adults but can also affect the pelvis.

**Methods:**

This single-center study is a retrospective review of 17 patients with primary ABCs of the pelvis. It examines the importance of polidocanol instillations as minimally invasive treatment option for ABCs of the pelvis compared to intralesional curettage or marginal resection.

**Results:**

Seventeen patients with the diagnosis of a primary ABC of the pelvis were included in the study. Six patients were male (35%) and 11 patients female (65%); the mean age was 18 (9-49) years. The mean follow-up time was 50 months (12-136 months). The most common location of manifestation was the pubis (6; 35%), followed by the ilium (6; 35%), the sacrum (3; 18%) and the ischium (2; 12%). Eight patients were treated by intralesional curettage with the use of adjuvants, one patient by marginal resection, seven by sequential instillation of polidocanol and one patient by simple observation. Five patients received an additional transarterial embolization. After intralesional curettage local recurrence was detected in 4/8 cases (50%). After instillation therapy six patients (86%) had a stable disease without recurrence, only one patient (14%) had a persistent disease with need of additional treatment and was therefore converted to intralesional curettage without local recurrence in the follow-up.

**Conclusions:**

Sequential instillations of polidocanol are a promising, minimally invasive treatment method for ABCs of the pelvis and can be well combined with transarterial embolization.

## Introduction

The aneurysmal bone cyst (ABC) is a benign but rapidly and aggressively growing tumor that occurs in adolescents and young adults. ABCs can appear in the whole skeleton but usually affect the metaphysis of long bones or the pelvis [1]. 70% of the cysts appear as primary lesions, 30% are secondary with preexisting osseous lesions [2].

Patients usually present with pain and swelling in the affected region and sometimes pathological fractures can occur. If the cyst expands progressively, it forms an expansile mass and may be visible or palpable. In plain radiographs an osteolytic, multilobulated lesion can be detected; the magnetic resonance imaging shows cystic formations with typical fluid-fluid levels due to blood sedimentation [3]. Two major classifications are used for distinguishing cystic lesions of the bone: the Enneking [4] and the Capanna [5] classification. ABCs have to be diagnosed by biopsy and histopathological examinations [6].

The pathophysiology of ABCs is debatable [2]. Recent cytogenetic and molecular studies underline the neoplastic character of ABCs due to of specific translocations of the ubiquitin-specific protease (USP) 6 gene [7]. USP6 gene arrangements are seen in 70% of primary ABCs and are lacking in secondary ABCs [8]. Histologically ABCs contain blood filled spaces surrounded by variable thick fibrous septae. Within the septae are uniform blad spindle cells, multinucleated giant cells, capillaries and varying amount of matrix findable [2]. By fluorescent in situ hybridization and next generation sequencing USP6 gene arrangements can be detected in up to 100% of the cases [9]. With these examinations primary ABCs can be differentiated from secondary ABCs and other bone affecting tumors. Other bone pathologies like a chondroblastoma, a giant cell tumor of bone, a chondromyxoid fibroma, a fibrous dysplasia or a telangiectatic osteosarcoma have to be excluded. In rare cases a malignant transformations of ABCs is reported [2].

Different treatment modalities are described in the literature and can be divided into nonoperative, minimally invasive and operative treatment options. Among those are wide resection, marginal resection, intralesional resection such as curettage with or without adjuvants, selective arterial embolization, intralesional sclerotherapy using polidocanol or the systemic application of denosumab [3]. The optimal treatment, especially in the pelvis is still under discussion. A wide surgical resection results in best local control rate but can evoke other complications depending on the dimension of the resection and the localization [10]. Especially in the pelvis a wide resection may cause severe restrictions on the functional outcome. Due to large soft tissue components of an ABC a marginal or wide resections can be difficult to achieve and the surgical approach has to be extensive. The most common therapy for ABCs is an intralesional curettage with the use of adjuvants. Adjuvants can be the use of a high-speed drill, phenol, hydrogen peroxide or cement augmentation with polymethylacrylat (PMMA). Novais et al. described intralesional curettage extended with a high-speed burr and bone grafting in a study with 13 patients with an ABC of the pelvis as sufficient way of therapy [11]. Even local MRI-guided percutaneous cryoablation has been used as adjuvant therapy [12]. Local recurrence rates after intralesional curettage of up to 50% are reported in the literature [13]. Transarterial embolization of ABCs can be used as adjuvant or single therapy when surgical resection is difficult or connected with high risk of complications. Henrichs et al. reported the successful use of transarterial embolization in ABCs of the sacrum as single therapy [14]. Sclerosants such as polidocanol can be used for instillation of the cyst and lead stepwise to a resolution of the cystic lesion and progressive sclerosis. Rastogi et al. reported a cure rate of 97% in a study with 72 patients with primary ABC [15]. The RANKL inhibitor denosumab can be used in cases when surgical procedures and/or embolization are insufficient or without effect.

### Purpose of the study

Several case reports and only one larger historic case series about the manifestation of ABCs in the pelvis and sacrum are findable in the literature [16–18]. The present study describes 17 cases of an ABC of the pelvis treated by intralesional curettage, percutaneous instillation of polidocanol and, in selected cases, arterial embolization. It comments on the question if sequential instillations of polidocanol are at least equally efficient in the treatment of ABCs of the pelvis compared to intralesional curettage or marginal resection.

## Patients and methods

The study protocol of this study was approved by the regional ethics committee (reference no.: 2019-592-f-S).

This single-center study is a retrospective review of 74 patients with a primary ABC, who were treated at a tertiary academic referral center for orthopedic oncology from 2009 to 2020. Among those, 17 ABCs were located in the pelvis or sacrum. No financial support was received for this study.

Epidemiological data, radiographic and histological examinations, different surgical techniques, complications and especially local recurrence were analysed. Imaging studies at time of presentation, including plain radiographs and MR-scans were reviewed in each case. The ABCs were classified radiologically by two systems: the Enneking classification and the classification according to Rastogi et al. [4, 5, 19]. The classification according to Rastogi et al. was exclusively used for the instillation group [19]. The diagnosis was made by histological and immunohistological examinations by expert pathologists. In addition to the standard histopathological examination, fluorescence in situ hybridization (FISH) and next generation sequencing were used to differentiate primary from secondary ABCs.

The following treatment options were performed: intralesional curettage with hydrogen peroxide as adjuvant and sequential instillation of polidocanol (Aethoxysklerol, 3%, Kreussler pharma; Wiesbaden, Germany). Intralesional curettage was performed when the manifestation of the cyst was predominantly restricted to the bone and the soft tissue component small. For defect reconstruction a synthetic calcium phosphate bone graft (Actifuse®; Baxter Deutschland; Unterschleißheim, Germany) or PMMA (Palacos®; Heraeus Medical; Wehrheim, Germany) were used. Sequential instillations of polidocanol (Aethoxysklerol® 3%; Kreussler pharma; Wiesbaden, Germany) were performed in cases where the surgical approach was difficult, a relevant soft tissue component was detectable, or lesions directly adjoined the acetabulum. Short- or long-term complications were reviewed. Patients underwent follow-up with clinical and radiographic examinations (radiographs and MR-scans) at 3-month intervals in the first 2 years and at 6-month intervals for the following 4 years.

Due to the low number of patients in the different treatment groups the statistical analysis was predominantly performed descriptively. Statistical analysis was performed with the use of SPSS Statistics (IBM Corp. Released 2019, Version 26.0. Armonk, NY, USA). Continuous variables like age and time of follow up were described using the mean and the maximum and minimum. Cyst volumes before and after treatment were analysed using the Wilcoxon test; local recurrence rates were compared using Fisher’s exact test.

## Results

This study includes 17 patients with the histopathological diagnosis of a primary ABC of the pelvis. Out of these, six patients were male (35%) and 11 patients female (65%; Table 1); the mean age was 18 (9-49) years. The mean follow-up time was 50 months (12-136 months). The most common location (Table 1) was the pubis (6; 35%), followed by the ilium (6; 35%), the sacrum (3; 18%) and the ischium (2; 12%). According to the Enneking [4] classification six lesions were rated as active and 11 as aggressive. The examined ABCs had a mean initial volume of 54 cm^3^ (8 – 223 cm^3^) in MRI scans.

**Table 1:** Overview of the study collective

	Gender	Age	Localization	Volume in cm^**3**^	Enneking classification	Rastogi classification	Treatment	Residual volume in cm^**3**^	Additional treatment	Recurrence/persistent disease	Necessity of treatment	Complications	Number of instilations	Second recurrence	Follow-up (months)
1	female	49	sacrum	8	aggressive	not applicable	intralesional curettage + PMMA	–	No	recurrence	no	none	–	no	11
2	male	13	ischiium	12	active	not applicable	intralesional curettage + PMMA	–	no	recurrence	no	none	–	no	28
3	female	15	pubis	38,1	aggressive	not applicable	intralesional curettage + bone substitute	–	no	no	no	none	–	no	36
4	female	18	ilium	11,9	active	not applicable	intralesional curettage + bone substitute	–	no	no	no	none	–	no	7
5	female	15	ilium	42	active	not applicable	biopsy + watch and wait	–	no	no	no	none	–	no	12
6	male	11	ischium	45,7	aggressive	not applicable	marginal resection and reconstruction with PMMA	–	no	persistent disease	no	healing disorder	–	no	23
7	female	12	sacrum	35	aggressive	not applicable	marginal resection	–	embolization	persistent disease	no	none	–	no	44
8	female	9	ilium	27,7	active	not applicable	intralesional curettage + bone substitute	–	no	recurrence	yes	none		yes	100
9	male	42	pubis	66,9	aggressive	not applicable	intralesional curettage + PMMA	–	embolization	recurrence	yes	none	–	no	136
10	male	11	ilium	222,6	active	not applicable	intralesional curettage + PMMA	–	embolization	persistent disease	yes	healing disorder	–	no	89
11	female	14	ilium	43,8	active	I	instillation of polidocanol	0	no	no	no	none	3	no	59
12	female	16	pubis	106	aggressive	I	Instillation of polidocanol	9,1	no	persistent disease	no	none	7	no	37
13	male	16	pubis	40,1	aggressive	I	Instillation of polidocanol	1,94	embolization	persistent disease	yes	none	7	no	50
14	male	16	pubis	20,2	aggressive	III	Instillation of polidocanol	14,69	no	persistent disease	no	none	8	no	51
15	female	16	pubis	56,7	aggressive	III	Instillation of polidocanol	31,27	embolization	persistent disease	no	none	6	no	58
16	female	10	ilium	113,3	aggressive	III	Instillation of polidocanol	58,98	embolization	persistent disease	no	none	10	no	62
17	female	16	sacrum	32,7	aggressiv	IV	Instillation of polydocanol	32,44	embolization	persistent disease	no	none	6	no	50

**Fig. 1:**
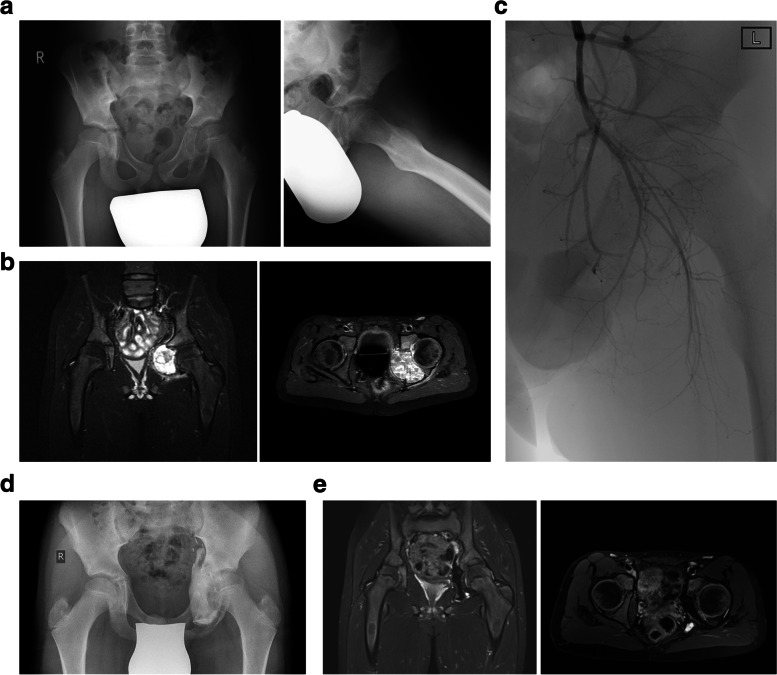
**a**-**e** Manifestation of an ABC in the left ilium and ischium: simple radiograph (**a**) and MRI scan (**b**); preoperative angiography without embolization (**c**); radiograph after intralesional curettage and cement augmentation (**d**), MRI scan 24 months after operation with small residual cystic element (**e**)

In eight cases an intralesional curettage (Fig. 1a-e) was performed. According to the Enneking classification four lesions of this subgroup were rated as active and four lesions as aggressive. The mean cyst volume in the curettage group was 51 cm^3^ (8-223 cm^3^). In one case an additional transarterial embolization was performed before curettage. In 50% (4/8 cases) a histologically confirmed local recurrence occurred after intralesional curettage. In one case of local recurrence a re-curettage was performed, in 3 cases a series of sequential instillations of polidocanol was started. The mean time until local recurrence arose was 63 (29-65) months. In two cases a wound healing disorder was observed in the curettage group. After treatment of recurrence, no second recurrence was observed in the study group.

**Fig. 2:**
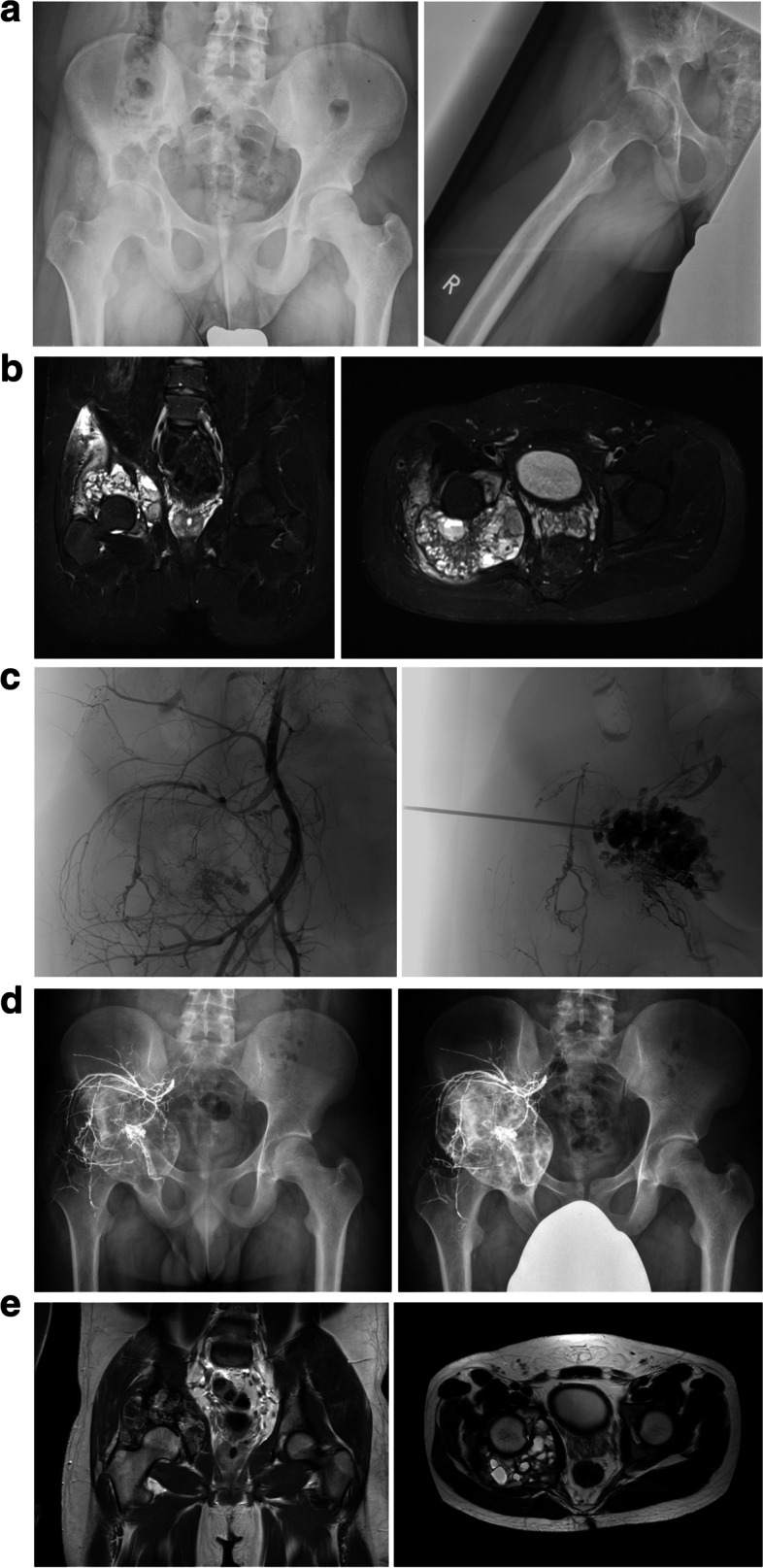
**a**-**e** ABC of the right ilium of a 15-year-old patient: simple radiograph (**a**); MRI scan (**b**); transarterial embolization and percutaneous sclerotherapy (**c**), simple radiographs 6 weeks (left) and 24 months after the sixth and last instillation (right; **d**); MRI scan 24 months after the last sclerotherapy with constant residual cystic elements (**e**)

In 7 cases a series of sequential polidocanol instillations (Fig. 2a-e) was performed as primary treatment. According to the Enneking classification one lesion of this subgroup was rated as active and six lesions as aggressive. The mean cyst volume in the instillation group was 59 cm^3^ (20-113 cm^3^). The mean number of instillations was 7 (3-10); the mean volume of polidocanol was 6 ml (2-10 ml). Transarterial embolization was performed in four cases. The mean cyst volume after instillation therapy was reduced significantly (*p* = 0.018) to 21 cm^3^ (0-60 cm^3^) in the sense of a persistent disease without need of additional treatment. In one case (14%) persistent cystic formations including typical fluid-fluid levels without progressive sclerosis of the cyst’s wall were observed. This case was classified as persistent disease with need of additional treatment and an intralesional curettage was performed. No local recurrence was observed after additional curettage. Mean duration until end of treatment in the instillation subgroup was 9 (1-23) months.

In one case of an ABC in the study group a marginal resection was performed. Due to a predominant soft tissue part and the good surgical accessibility this treatment option was chosen. No local recurrence was detected in this case. One further case did not receive any kind of surgical treatment but simple observation due to an asymptomatic course of disease.

## Discussion

The ABC is a rare, benign, but locally aggressive bone tumor with a high rate of local recurrence. Diagnosing an ABC is often challenging, the biopsy is compulsory, and the results of the radiologic and histopathological examinations have to be seen in synopsis with the clinic history. A differentiation between primary and secondary ABCs is important and highly relevant for an adequate treatment.

This study describes the results of three common treatment options of ABCs at the pelvis: Intralesional curettage, sequential percutaneous instillation of polidocanol and marginal resection. Sex distribution (*p* = 0.41), age (*p* = 0.81) and pre-interventional MRI volume (*p* = 0.3) did not differ significantly in the curettage- and instillation subgroup. Only one patient was treated by a marginal resection; instillation and curettage were each used in equally limited numbers of cases. Our study showed a local recurrence in 4/8 cases (50%) treated by intralesional curettage. Regarding ABCs of the pelvis Panayiotis et al. [18] described a local recurrence in 14% of the cases in a historic study cohort of 35 patients treated by excision-curettage or intralesional excision between 1921 and 1996. Novais et al. [11] reported an equally low recurrence rate of 8% in a study collective of 13 patients who were treated by intralesional curettage. Among these patients five were treated by transarterial embolization preoperatively. Larger studies on patients with primary ABCs in various locations described local recurrence rates of more than 40% after the use of intralesional curettage [2, 3, 13]. Especially in the pelvis, where intralesional curettage is more challenging due to the difficult surgical approach, low local recurrence rates of less than 15% are hardly achievable.

In the instillation subgroup 6/7 (86%) patients were treated successfully by instillation therapy. 1/7 cases (14%) with a persistent disease needed further treatment in terms of an intralesional curettage. After a combination of polidocanol instillations and additional intralesional curettage no recurrence was observed. Rastogi et al. [15] demonstrated the good response of primary ABCs to polidocanol instillation in a series of 72 patients with primary ABCs: A reduction of the initial cyst volume to 25% or less was achieved in 66.7% of the cases. Overall, 30.5% of Rastogi et al.’s collective showed a residual volume of 25-50% after treatment. In our study the mean initial cyst volume decreased significantly from 59 to 22 cm^3^ (*p* = 0.018) after polidocanol instillation. In three cases (3/7;42.9%) a cyst volume reduction to 25% or less of the initial volume was achieved. Rastogi et al. classified “inadequate healing” as a residual cyst volume of more than 50% of the initial volume and reported this in 2.8% of the cases only [15]. The present study showed a residual volume of 50% or more in 57% (4/7) of the patients. The significant difference concerning adequate and inadequate healing in both studies may be explainable by (1.) the lower initial cyst volume of Rastogi’s collective (26.1 cm^3^) and (2.) the low number of ABCs in the pelvis (*n* = 3). Several other studies described a single instillation of polidocanol as sufficient in treatment of ABCs [15, 20, 21]. In our study the mean number of instillations was 6.7 and not a single patient was treated by a single instillation sufficiently. Rastogi et al. described a mean number of three instillations but even about a successful healing process after a single instillation in rare cases [15]. Masthoff et al. [22] showed the successful treatment of 16 patients with refractory and nonresectable ABCs by transarterial embolization in addition to percutaneous sclerotherapy. In 9/16 cases the ABCs were located in the pelvis. A mean of 1.6 transarterial embolizations and 3.2 percutaneous sclerotherapies were performed. No recurrence was seen in a median follow-up time of 27.3 months. The present study gives consent to other authors concluding that the percutaneous instillation of polidocanol is a less invasive procedure than intralesional curettage [23].

Varshney et al. [23] reported about a comparative study of 94 patients with a primary ABC who were treated by intralesional curettage or sequential percutaneous instillation of polidocanol. No significant differences for recurrence rates between the two treatment groups were found by the authors. They conclude that the less invasive character of the instillation therapy justifies it as primary attempt of treatment. In the present study the comparison of local recurrence rates with necessity of treatment for the instillation subgroup (4/8; 50%) and the curettage group (1/7; 14%) did not show a statistically significant difference (*p* = 0.57) either. However, in a larger study with a higher number of patients the statistical analysis may become significant.

In conclusion, our results along with the aforementioned studies show that the percutaneous instillation of polidocanol is a less invasive procedure than intralesional curettage and is mostly favorable for cystic lesions in the pelvis due to its minimally invasive character. Moreover, we see advantages of the instillation therapy as a treatment of ABCs with a high initial cyst volume and for cystic lesions in locations with a difficult surgical approach. Percutaneous instillation can be well combined with transarterial embolization and can be performed in general or local anesthesia in an outpatient clinic without hospitalization.

In the present study no patient received denosumab, however, denosumab can be used in cases when surgical procedures and/or embolization are not successful or applicable. Lange et al. used denosumab in two cases of ABC of the spine where embolization failed [24].

This single-institution series has severe limitations due to its retrospective, observative character and the very low number of patients. The patients and compared groups were not randomized and predominantly descriptive statistical analysis was conducted. Despite the limitations the present case series presents useful descriptive information for the diagnosis and treatment of ABCs of the pelvis.

## Conclusion

Sequential percutaneous instillations of polidocanol combined with transarterial embolization are a relevant treatment option for ABCs of the pelvis. The less invasive character of the instillation justifies it as preferable therapy, especially for borderline resectable lesions. In this small cohort ABCs have a tendency to lower local recurrence after sequential instillations of polidocanol than after intralesional curettage. Nonetheless, several instillations can be necessary. In some cases, a conversion to intralesional curettage may be inevitable.

## Data Availability

The datasets used and/or analysed during the study are available from the corresponding author on reasonable request.

## Abbreviation

ABC: Aneurysmal bone cyst
